# AZ31 Magnesium Alloy Foils as Thin Anodes for Rechargeable Magnesium Batteries

**DOI:** 10.1002/cssc.202101323

**Published:** 2021-08-31

**Authors:** Ananya Maddegalla, Ayan Mukherjee, J. Alberto Blázquez, Eneko Azaceta, Olatz Leonet, Aroa R. Mainar, Aleksey Kovalevsky, Daniel Sharon, Jean‐Frédéric Martin, Dane Sotta, Yair Ein‐Eli, Doron Aurbach, Malachi Noked

**Affiliations:** ^1^ Department of Chemistry Bar Ilan University Ramat Gan Israel; ^2^ CIDETEC Basque Research and Technology Alliance (BRTA) P Miramón, 196 Donostia-San Sebastián 20014 Spain; ^3^ Israel Institute of Metals Technion R&D Foundation Technion City Haifa 3200003 Israel; ^4^ Institute of Chemistry The Hebrew University of Jerusalem Jerusalem 9190401 Israel; ^5^ Univ. Grenoble Alpes, CEA, Liten, DEHT Grenoble 38000 France; ^6^ Department of Materials Science and Engineering Technion-Israel Institute of Technology Haifa 3200003 Israel; ^7^ Grand Technion Energy Program (GTEP) Technion-Israel Institute of Technology Haifa 3200003 Israel

**Keywords:** batteries, electrochemistry, energy storage, Mg electrodes, rechargeable Mg batteries

## Abstract

In recent decades, rechargeable Mg batteries (RMBs) technologies have attracted much attention because the use of thin Mg foil anodes may enable development of high‐energy‐density batteries. One of the most critical challenges for RMBs is finding suitable electrolyte solutions that enable efficient and reversible Mg cells operation. Most RMB studies concentrate on the development of novel electrolyte systems, while only few studies have focused on the practical feasibility of using pure metallic Mg as the anode material. Pure Mg metal anodes have been demonstrated to be useful in studying the fundamentals of nonaqueous Mg electrochemistry. However, pure Mg metal may not be suitable for mass production of ultrathin foils (<100 microns) due to its limited ductility. The metals industry overcomes this problem by using ductile Mg alloys. Herein, the feasibility of processing ultrathin Mg anodes in electrochemical cells was demonstrated by using AZ31 Mg alloys (3 % Al; 1 % Zn). Thin‐film Mg AZ31 anodes presented reversible Mg dissolution and deposition behavior in complex ethereal Mg electrolytes solutions that was comparable to that of pure Mg foils. Moreover, it was demonstrated that secondary Mg battery prototypes comprising ultrathin AZ31 Mg alloy anodes (≈25 μm thick) and Mg_*x*_Mo_6_S_8_ Chevrel‐phase cathodes exhibited cycling performance equal to that of similar cells containing thicker pure Mg foil anodes. The possibility of using ultrathin processable Mg metal anodes is an important step in the realization of rechargeable Mg batteries.

## Graphical abstract

**Ultra‐thin Mg alloy**: Rechargeable magnesium batteries are considered as the most promising alternative to lithium battery technologies Similar electrochemical performance of 100 μm thick pure Mg foils and 25 μm thin and ductile AZ31 Mg alloy foils as anodes in rechargeable Mg batteries is demonstrated. Comprising Chevrel‐phase (Mo_6_S_8_) cathodes, thin foils of AZ31 Mg alloy can serve as very suitable anodes in rechargeable Mg batteries.

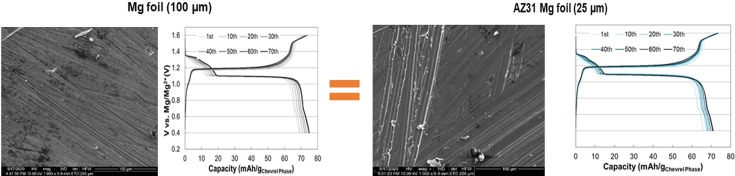

## Introduction

The extensive demand for rechargeable lithium‐ion batteries (LIBs) leads scientists and engineers to search for alternative rechargeable battery chemistries.^[1]^ Rechargeable magnesium batteries (RMBs) are considered as one of the most promising post‐LIB technologies.[^2^, ^3^, ^4^] The abundance of Mg in the Earth crust is orders of magnitude higher than that of Li, which may make RMBs a much more cost‐effective battery technology than LIBs.^[5]^ Due to its bivalency, Mg has particularly high volumetric capacity (3833 mAh cm^−3^), higher than that of Li metal (2046 mAh cm^−3^). In addition, unlike Li metal, Mg is much less prone to uncontrolled growth of metallic dendrites during electrodeposition, so the operation of a Mg metal‐based battery is much safer compared to Li metal batteries.

Various RMB prototype systems were proposed and showcased throughout the last two decades; however, the commercialization of these systems is still far from realization. The challenges facing RMB consist of poor durability and compatibility of the cell components (cathode, anode, and electrolyte solution) with the intense chemical and electrochemical conditions that are required for any rechargeable battery operation. One of the biggest hurdles in the operation of metal anode‐based batteries such as RMB is to obtain a reversible metal deposition at the anode side.^[6]^ Most aprotic solvents and salts relevant to the field of batteries react with Mg metal to form passivation layers that contain species such as MgO, MgCO_3_, and Mg(OH)_2_.^[6]^ Passivating layers comprising ionic Mg compounds impede the necessary transport of Mg ions at the electrode‐electrolyte solution interface and therefore may prevent any reversible behavior of Mg electrodes. Another drawback that limits the practicability of RMB are poor mechanical properties of pure Mg foils, with a tensile strength of around 20 MPA as well as low ductility (0.12–0.2)^[7]^ and high brittleness of thin Mg foils. These poor mechanical properties may interfere significantly with mass production of such metal anode‐based battery systems.^[8]^ Another challenge in the field is elaboration of electrolyte solutions with a wide enough electrochemical window, in which Mg anodes behave fully reversibly. Only ether solvents seem to be relevant for RMBs because they are not reactive with Mg metal, can dissolve Mg salts/complex electrolytes that enable reversible behavior of Mg metal anodes, and exhibit wide electrochemical windows (anodic stability >3 V vs. Mg). During the last decades, a number of ethereal solutions relevant for RMBs were developed. Some of them are based on organometallic complexes, and there are also ethereal solutions containing conventional Mg salts within this category. For instance, solutions comprising dimethoxy ethane (DME), Mg(N(SO_2_CF_3_)_2_)_2_ (MgTFSI), and MgCl_2_ enable reversible behavior of Mg metal anodes and exhibit high anodic stability that may fit a variety of relevant cathodes. This specific solution cannot be our choice for the present study because it requires a special pretreatment (cleaning from unavoidable atmospheric contaminants like trace water and oxygen) in order to enable fully reversible Mg deposition/dissolution processes. More suitable are solutions containing organometallic complexes that react readily with contaminants in ethereal solutions, neutralize them, and hence, do not require any pretreatment after their preparation. Most suitable are the so‐called APC (all phenyl complex) solutions, which are prepared by interacting C_6_H_5_MgCl and AlCl_3_ in THF. The solutions thus formed include Mg(5THF)Cl^+^ and Mg_3_Cl_3_(6THF)^+^ cations and AlCl_4‐*x*_(C_6_H_5_)_*x*_^−^ anions, allowing fully reversible behavior of Mg metal anodes and exhibiting a very impressive anodic stability (>3 V vs. Mg).[^9^, ^10^] Thereby, they are very suitable for the studies described herein.

In order to reach high energy density with Mg batteries, the use of very thin Mg metal foils anodes is mandatory. However, here the low ductility of pure Mg metal makes the preparation of very thin Mg foil electrodes difficult. The best approach to overcome the poor mechanical properties of pure Mg metal foils is to use Mg alloys, containing different elements in small amounts.[^11^, ^12^, ^13^, ^14^] Considering that alloys might have lower chemical activity than pure metals, the use of metal alloy electrodes instead of pure metal electrodes in electrochemical devices requires rigorous compatibility tests. In the present case, the behavior of Mg alloy anodes in electrolyte solutions in which Mg deposition is supposed to be reversible must be rigorously explored. Alloying can help in tuning the mechanical and structural properties of the anode material, as well as improving their processing capabilities.^[15]^ However, Mg alloys with high concentration of elements such as Al, Bi, P, Si, and Sn has inherent drawbacks when compared to pure Mg anodes. First, the high concentration of the alloying elements may adversely affect the main strength of a metal battery system, its high energy density. In addition, the large volume changes during the alloying/dealloying processes can result in structural deformations of the anodes during extensive cycling. In turn, using low enough concentrations of foreign elements in alloys can assist in keeping the structural integrity of the anodes during prolong cycling, yet affecting very positively their ductility and flexibility.[^16^, ^17^] Moreover, using Mg alloys anodes with low concentration of foreign elements may not worsen their electrochemical behavior in ethereal electrolyte solutions that were found suitable for RMBs.

In this study, we assessed the possibility of using a Mg alloy with low concentrations of foreign elements as a source for thin metallic foil anodes for RMB. One of the challenges in processing bulk magnesium alloy foils is their corrosive nature.[^18^, ^19^] The rapid oxidation of molten Mg in air along with the pyrophoric nature of Mg powders are additional problems that somewhat complicate manufacturing of Mg alloys for standard applications. Herein, by using suitable processing conditions, we fabricated ultrathin (≤100 μm) AZ31 Mg alloy (3 % Al; 1 % Zn) foils as anodes for RMB applications. The ultra‐thin AZ31 Mg alloy film drastically reduces the overall weight of a full Mg battery. AZ31 is a commonly used magnesium alloy with good room‐temperature strength and ductility (0.18–0.55)^[20]^ combined with corrosion resistance and weldability.[^21^, ^22^] We investigated the electrochemical performance of AZ31 Mg alloy foil electrodes in APC‐based electrolyte solutions and compared the results to those obtained in similar experiments with pure Mg foil electrodes. The experiments included tests of anodes alone and of full cells comprising Mg or Mg alloy anodes and Chevrel‐phase (CP) cathodes. We were pleased to realize that thin AZ31 Mg foil electrodes can be considered as compatible anodes for RMBs.

## Results and Discussion

**Figure 1:**
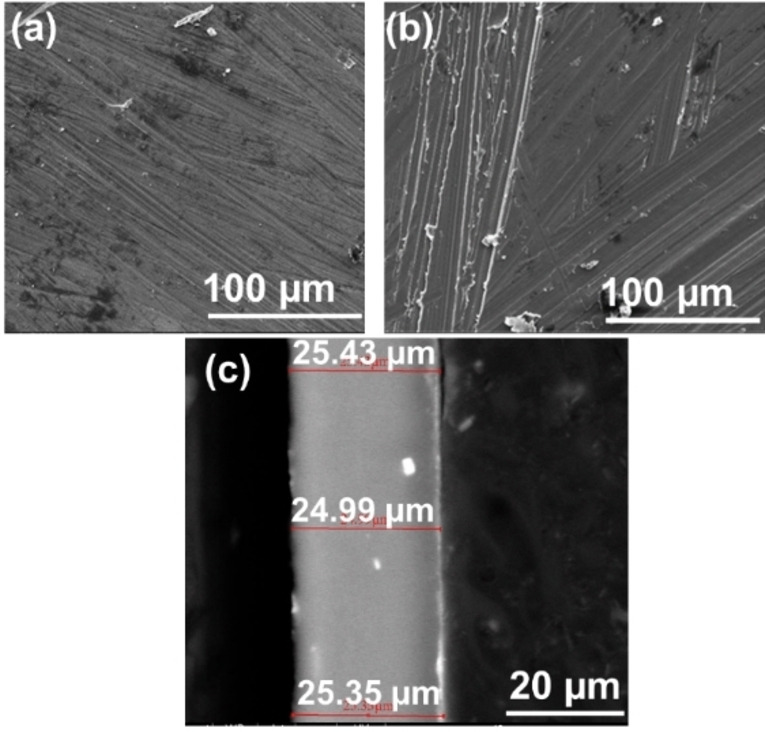
SEM images of the surface of pristine (a) Mg metal foil, (b) Mg alloy AZ31 thin film, and (c) cross‐section SEM image of ultrathin AZ31 foil.

In this study we examined the option of using thin AZ31 Mg alloy foils as anodes for RMBs. Due to the high ductility of the AZ31 Mg alloy, foils can be rolled under pressure to ultrathin thickness (≤100 μm) without forming significant fractures. Considering the lack of available database regarding rolling of AZ31 Mg foils, the degree of pressing by the rolls was determined experimentally. We found that limiting the thickness decrease to 6–7 % during each individual rolling step resulted in a uniform and crack‐free thin AZ31 foils, which is an evidence of a properly adjusted rolling process. The top‐down scanning electron microscopy (SEM) images of the pure Mg metal and AZ31 thin film surfaces of thickness 25 μm are presented in Figure 1a, b, respectively. The SEM image in Figure 1c and Figure S1 presents the cross‐section of the 25 and 100 μm AZ31 foil. As can be seen, the sequential rolling procedure results in small thickness distribution of the AZ31 foil. We can see that the surface morphology and topography of both pristine electrodes are pinholes and crack free. We note that the scratches on the metal surfaces are a result of native oxide layer (MgO) removal using sharp glass slides.

**Figure 2:**
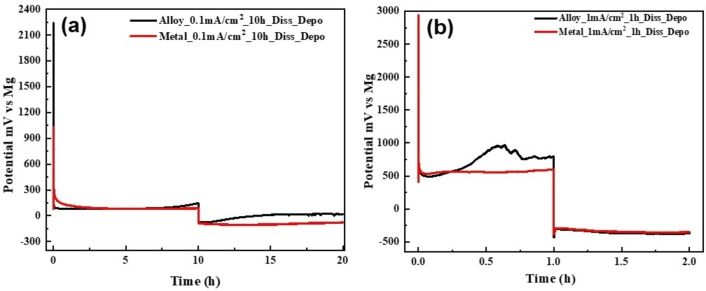
Voltage profile of dissolution‐deposition process on AZ31 alloy thin film and Mg metal thin film as anodes at current densities of (a) 0.1 mA cm^−2^ for 10 h and (b) 1 mA cm^−2^ for 1 h, in 0.25 m APC/THF solution, with Mg metal as counter and reference electrodes. The charges involved in these processes were 1 mA and 0.1 mA per cm^2^, respectively.

**Figure 3:**
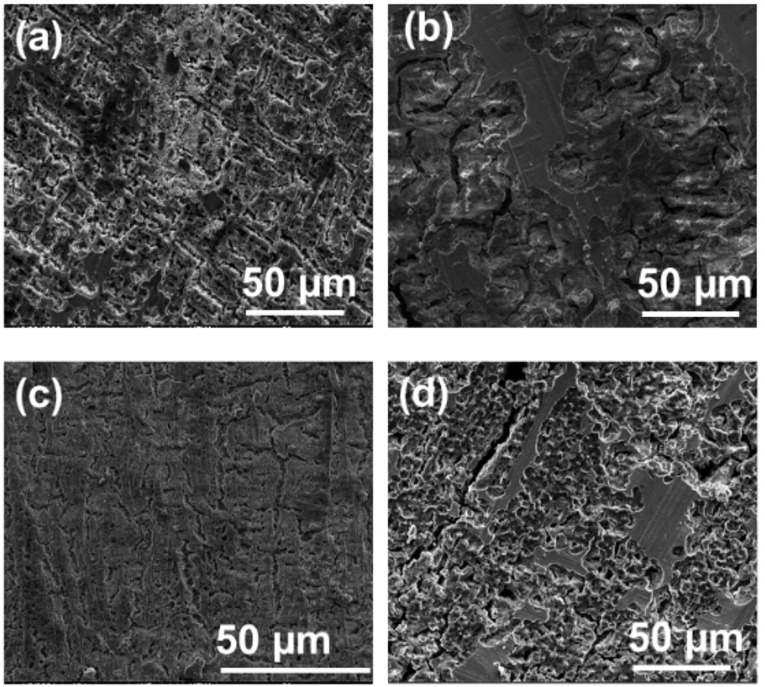
SEM images of electrodes after dissolution processes. Images a and b relate to pure Mg foil electrodes after dissolution at 1 mA/cm^2^ for 1 h and 0.1 mA/cm^2^ for 10 h, respectively. Images c and d relate to AZ31 Mg alloy foil electrodes after dissolution at 1 mA/cm^2^ for 1 h and 0.1 mA/cm^2^ for 10 h, respectively. 0.25 M APC/THF solutions.

The discharge and charge voltage profiles of the AZ31 and pure Mg anodes at different current densities are presented in Figure 2. At low current density of 0.1 mA cm^−2^, both the alloy and the pure Mg exhibit stable dissolution and deposition voltage profiles for a duration of 10 h each, with a low overpotential (*η*=±100 mV vs. Mg metal; Figure 2a). The long‐term (40 h) dissolution/deposition cycling process at 0.1 mA cm^−2^ (Figure S2) exhibits similar behavior. At relatively high current density of 1 mA cm^−2^ the two anodes present high overpotential (*η*=±500 mV vs. Mg metal) during the dissolution and the following deposition process for a duration of 1 h each (Figure 2b). We also notice that the dissolution voltage profile of the AZ31 Mg alloy anode is noisy than the voltage profile of the pure Mg foil electrode. Canepa et al.^[23]^ proposed that the Cl ions of the APC electrolyte are adsorbed on the metallic anode surface, readily exhibiting high exothermic adsorption energy and delaying Cl species adsorption on the anodic surface after charge transfer, affecting the deposition process. Moreover, to continue deposition of Mg metal at the anodic surface, the Cl ions, which accounted for deposition overpotential, should be removed continuously. The presence of AlCl_3_ in the electrolyte facilitates to keep the anode surface free of Cl, which improves the deposition kinetics. During the deposition process, the continuous removal of Cl ions accounted for high overpotential at the anode interface, but the presence of Cl ions facilitates the dissolution kinetics. During the dissolution process, the (MgCl)^+^ species are driven towards the cathode surface due to the applied potential, which significantly increases the local concentration of MgCl_2_ species at the anodic surface that dissociates further into (MgCl)^+^ and Cl^−^, generating more carriers to facilitate the reaction. Hence, an asymmetry arises between dissolution and deposition kinetics, leading to increase in overpotential. Nonetheless, we can conclude that the chrono‐potentiometric response of the pure Mg and AZ31 Mg alloy electrodes are very similar at low and high current densities. SEM Images of pure Mg and AZ31 Mg alloy electrode surfaces after first dissolution processes at different current densities are presented in Figure 3. As can be observed, all the samples present structural changes with respect to the smooth surface of the uncycled electrodes presented in Figure 1. Moreover, we can see that the type of the morphology change is a function of the current density. Figure 3a,b presents the morphology of pure Mg metal anode after the first dissolution process that was carried out at 0.1 mA cm^−2^ for 10 h and 1 mA cm^−2^ for 1 h. While some parts of the Mg surface are smooth, other areas present inhomogeneous rough topography. Similar dissolution behavior was observed for the AZ31 Mg alloy electrodes after 0.1 mA cm^−2^ dissolution process for 10 h, except fewer smooth areas were detected for these electrodes when compared to the pure Mg anodes. We note that, because of the low thickness of the foils that were used in this study, some areas were slightly punctured throughout the dissolution and deposition processes. The presence of smooth unreacted parts implies that the Mg dissolution process at the electrodes surface was not uniform, which led to the formation of uneven topography. At high current densities of 1 mA cm^−2^, the Mg dissolution is more uniform for both the alloy and the pure Mg anodes.

The images obtained with pure Mg anode surfaces after dissolution at 1 mA cm^−2^ show that most of the original surface was dissolved, while only negligeable areas were not activated during this process. The images related to AZ31 Mg alloy electrodes reflect a more uniform surface than that of pure Mg electrodes, what implies that their Mg dissolution process was more homogenous. The microcracking on the AZ31 Mg alloy electrodes seen in Figure 3c, d might correspond to structural stress that exists in the ultrathin metallic foils. We can conclude that the overall dissolution behavior of the AZ31 Mg alloy and the pure Mg foils is generally similar, with the AZ31 Mg alloy electrodes presenting a more uniform Mg dissolution at high current densities.

**Figure 4:**
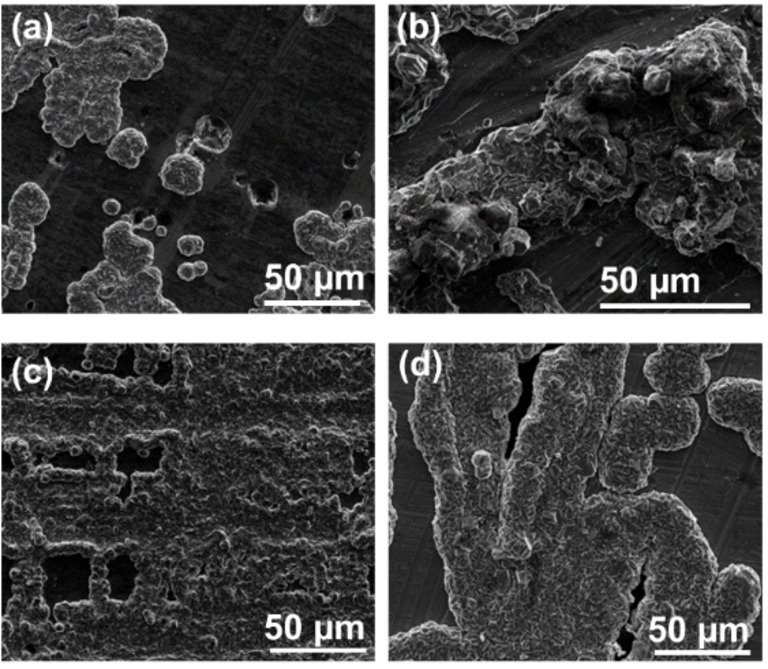
SEM images of Mg electrodes after deposition processes at different current densities. Images a and b relate to pure Mg anodes 1 mA/cm^2^ and 0.1 mA/cm^2^ respectively. Images c, d relate to AZ31 Mg alloy electrodes, −1 mA/cm^2^ (d) 0.1 mA/cm^2^, in 0.25 M APC/THF. The charge involved in these processes were 1 mA and 0.1 mA per cm^2^ respectively.

The SEM images of the following deposition process on the pure Mg and AZ31 Mg alloy electrodes at different current densities are presented in Figure 4. The surfaces of the pure Mg and AZ31 Mg alloy electrodes after deposition contain mainly Mg hexagonal crystals, which are the most common morphology in the Mg cells containing APC solutions.^[24]^ The size of hexagonal Mg deposits at current density of 1 mA cm^−2^ (Figure 4a) are larger than the crystals that were formed at higher current density of 0.1 mA cm^−2^ (Figure 4b). We can also observe that some amorphous deposits were formed on the Mg surfaces; these types of undefined deposits might correspond to adsorption and degradation of Mg−Al and Mg−Cl moieties.[^6^, ^23^]

Like the situation with pure Mg foil electrodes, the deposition process on AZ31 Mg alloy electrodes results in the formation of crystalline Mg on their surfaces. At a current density of 1 mA cm^−2^ (Figure 4c) the deposition is much more uniform on the AZ31 Mg alloy electrodes than on the pure Mg foil electrodes. Moreover, like the pure Mg sample, the deposition is less uniform at lower current densities of 0.1 mA cm^−2^ (Figure 4d). The higher deposition uniformity at higher current densities is a unique phenomenon for Mg electroplating in these nonaqueous complex solutions.[^25^, ^26^] It appears that the deposition of Mg from complex Mg cations solvated by both ether molecules and chloride anions in solutions like APC solutions can result in local changes in the solution activity, which temporarily increase the surface overpotential. As a result, the solvated Mg species preferentially deposit at alternative locations where the overpotential is smaller.^[25]^ Such situations may increase non‐uniformity as the current density is higher. In turn, high current density can also affect the diffusion and migration of the Mg cation complexes to/from the surface in a way that induces more uniform dissolution/deposition processes. Nonetheless, the most important observation is that the deposition trends and morphologies of the AZ31 Mg alloy and the pure Mg electrodes are comparable.

The elemental analysis of the anodes after deposition and dissolution processes was measured by X‐ray fluorescence (XRF). Table 1 summarizes the atomic percentages of Mg, Al, and Cl elements on the surfaces of the pure Mg and AZ31 Mg alloy anodes after dissolution and deposition processes at different current densities. As can be expected, the pristine AZ31 Mg alloy surface contains primarily Mg (96 %) and about 4 % of additional metals (Zn and Al), while the pure Mg foils contain only Mg on their surface. After the dissolution process at 0.1 mA cm^−2^ the percentage of Mg is decreased in favor of mainly Al and small amounts of Cl, in both the AZ31 Mg alloy and the pure Mg anodes. The Al and the Cl correspond to species that can be originated from residual complexes from the APC solution that were not washed out properly or from reactions between the complexes and the Mg metal surface, which include reduction of aluminate species to metallic aluminum deposits. We note that the decrease in the Mg content at the end of the dissolution process for the pure Mg and the AZ31 Mg anodes is very similar. After the deposition process, the Mg content increases with respect to the dissolution process, while the Al and Cl content is decreasing for both the pure Mg and AZ31 Mg alloy electrodes. We can also observe that the highest percentage of Mg (99.5 %) was found on the surface of AZ31 Mg alloy electrodes after being charged (Mg deposition process) with a current density of 1 mA cm^−2^. This high percentage of Mg after the charge process agrees with the uniform Mg deposition that took place on the AZ31 Mg alloy electrode surfaces (Figure 4c). The XRF analysis implies that the chemical composition of the pure Mg and AZ31 Mg alloy anode surfaces after the dissolution/deposition processes is highly similar. In summary, we can conclude that the deposition/dissolution processes of the AZ31 Mg alloy and pure Mg anodes were comparable. In the next section we discuss the behavior of the two types of Mg anodes when they are coupled with cathodes in full cells.

**Table 1:** Atomic percentage of Mg, Al, and Cl elements of pure Mg and AZ31 Mg alloy anodes at different current densities and state.

Material	Current density	Pristine [%]			Dissolution [%]			Deposition [%]		
	[mA cm^−2^]	Mg	Al	Cl	Mg	Al	Cl	Mg	Al	Cl
pure Mg	0.1	99.9	–	–	93	7	0.1	97.8	2	0.2
	1				87	12	0.5	97.8	2	0.2
AZ31	0.1	95.8	2.7	–	88	10.5	0.7	90	9.5	0.7
	1				86	11.5	2	99.5	0.1	0.1

**Figure 5:**
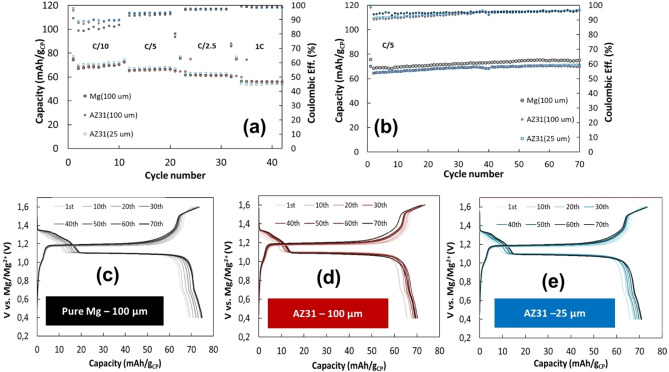
Measurements of full Mg cells, with Chevrel phase Mg_x_Mo_6_S_8_ (0<×<2) cathodes, (a) rate performance and (b) cycling performance of Mg (100 μm), AZ31 (100 μm) and AZ31 (25 μm), galvanostatic profile of various cycles of (c) Mg (100 μm), (d) AZ31 (100 μm) and (e) AZ31 (25 μm) in 0.25 M APC/THF solutions.

To evaluate the behavior of the two types of anodes in full Mg battery prototypes, we fabricated cells containing Mo_6_S_8_ CP Mg cathodes that were coupled to either pure Mg or AZ31 anodes with a thickness of 100 μm. To investigate the effect of the anode thickness on cell performance during prolong cycling, we also measured full cells with ultrathin 25 μm thick AZ31 Mg alloy anodes. Figure 5a presents the charge‐discharge rate performance of the three types of cells. The average discharge capacities at C/10, C/5, C/2.5, and 1C were 70, 65,60, and 55 mAh g_CP_^−1^, respectively. This decrease in capacity with increased current density is natural and expected, related to the trivial kinetic limitations of all the electrochemical reactions involved (Mg ions intercalation/de‐intercalation, solution ions transport, interfacial charge transfer resistances for Mg deposition/dissolution). It is highly important to realize that all the cells studied exhibit a very similar rate capability. An average decrease of around 21 % in the specific capacity per gram of CP cathode when increasing the rates in an order of magnitude (from C/10 to 1C) can be considered as a very good result for these Mg battery prototypes. We can also observe that the coulombic efficiency is increasing with C‐rates. Variations of the cell cycling efficiency, which increases at higher rates, can arise from several reasons, discussion of which is not important herein. It is important though that the behavior of all the cells in this respect is also very similar. Figure 5b presents the charge‐discharge capacity and coulombic efficiency of such cells (CP cathodes) with pure Mg and AZ31 Mg alloy anodes upon galvanostatic cycling with a constant current density of 10 mA g_CP_^−1^ (C/5 rate). We can observe that both types of cells present a stable capacity behavior during 70 cycles. The average discharge capacity of cells with pure Mg anode (70 mAh g_CP_^−1^) is slightly higher than that of cells with AZ31 Mg alloy anodes (65 mAh g_CP_^−1^). The coulombic efficiency of all the cells continually increases and stabilizes at around 98.8 % after 30 cycles. The initial irreversible loss in capacity can be associated with parasitic reactions such as electrolyte solution decomposition. Selected charge‐discharge voltage profiles of Mg (100 μm), AZ31 (100 μm), and AZ31 (25 μm) at C/5 are presented in Figure 5a–c respectively. All cells tested in this study present very similar voltage profiles typical for Mg ions intercalation/deintercalation processes with CP cathodes at room temperature.^[24]^ Hence, the most important outcome of this study of full cells is that their cyclability, rate capability, and cycling efficiency as a function of the experimental conditions are similar. These results are encouraging since they reflect very well the compatibility of the AZ31 Mg alloy thin foils to serve as anodes in RMBs.

## Conclusions

In this study we examined the feasibility for replacing commonly used pure Mg foils with AZ31 Mg alloy foils (3 % Al; 1 % Zn) as very thin anodes in rechargeable Mg batteries (RMBs). We found that the electrochemical and surface chemistry behavior of AZ31 Mg alloy thin foil anodes during Mg dissolution and deposition process is very comparable to that of pure Mg foil anodes. The morphology and surface topography after Mg dissolution or deposition processes are even more uniform with AZ31 Mg alloy than with pure Mg anodes. Interestingly, the uniformity of these processes with the Mg alloy anodes increases as their current density is higher. An excellent compatibility of thin (25 μm) AZ31 Mg foils as anodes was also realized in tests with full cells, comprising Chevrel‐phase (Mo_6_S_8_) cathodes. We can conclude that thin foils of AZ31 Mg alloy can serve as very suitable anodes in RMBs. This conclusion has a very important practical significance since the ductility of this Mg alloy is much better than that of pure Mg metal. These means that very thin Mg anodes with excellent mechanical properties and integrity can be prepared in relatively easy and straightforward industrial processes, implying a cost‐effectiveness as well. The possibility to use as thin Mg foil anodes as possible in RMBs is critically important for extracting high energy density from such advanced devices.

## Experimental Section

### Materials

Anhydrous AlCl_3_ (99.999 %), tetrahydrofuran (THF), and phenyl magnesium chloride in 2.0 m THF solution were purchased from Sigma Aldrich. THF was dried inside the glovebox with activated 4 Å molecular sieves for at least 72 h. Platinum foil (10×10×0.1 mm; 99.95 %) was purchased from Holland Moran. Pure Mg foil (0.10×50×50 mm) was purchased from NewMet Ltd. All sample preparations and electrochemical measurements were performed inside an Ar‐filled glovebox (Siemens), with water and moisture levels below 1 ppm.

### Electrolyte solution synthesis

To prepare 0.25 m APC solution, 0.25 m AlCl_3_ was added slowly into 0.5 m PhMgCl/THF solution. The resulting electrolyte solution was stirred at room temperature for 24 h inside the glovebox.

### Magnesium alloy foils processing

For the electrode preparation, 0.1 mm thick chunks of AZ‐31 Mg alloy (20 mm wide and 100 mm) long were purchased from Hunan High Broad New Material Co. Ltd. They were rolled further in order to form thin foils. The rollers of the rolling machine were heated to a temperature of 250 °C. The rolling procedure is shown in Figure S3. Samples were also heated to this temperature in a furnace located near to the rolling machine. The samples were clamped between the rollers, rolled, and placed back into the oven. The rotation speed of the rollers was 4–6 turns per minute, and the pressing force was controlled by the distance between the rollers. The clamping force of the rollers was changed, and the process was repeated. As a result, the foils were thinned to a thickness of 20–25 microns (Figure S4), while their length and width also increased to 250 and 30 mm, respectively. Chemical composition of samples before and after rolling is presented in the Tables S1 and S2, respectively. The chemical composition of the sample after rolling fully corresponds to the standard chemical composition of the AZ31 alloy.

### Electrochemical characterizations

The detailed preparation of the cathode composite for electrochemical cycling is described in the Supporting Information. The electrochemical experiments were carried out using a VPS‐300 multichannel potentiostat/galvanostat system from Bio‐logic Co. Electrochemical measurements were performed in 3‐electrode flooded cells at room temperature, with magnesium foils as both counter and reference electrodes (very wide and very narrow ones, respectively) for all the experiments. The native surface layer was removed from all electrodes in the glovebox, and then the electrodes were washed with dry THF before the experiments. All the working electrodes were also washed with dry THF after the experiments to remove residue of the electrolyte from their surface before taking them for post‐mortem analyses.

Electrochemical characterization of Mg metal and AZ31 alloy thin films anodes was performed through half cells (vs. Mg counter electrodes) and in full cells vs. CP cathodes in BaSyTec coin cells at 25±1 °C and different rates (C/10, C/5, C/2.5, and 1C). The cycle life of the coin cells was also investigated at C/5 rates charge‐discharge current rate within a 0.4–1.6 V interval.

### Structural and elemental analysis

The surface morphology of electrodes after electrodeposition and stripping processes was studied by SEM (Quanta 2000, from FEI). Elemental analysis was performed using XRF measurements and inductive coupled plasma atomic emission spectrometry (ICP‐AES). For XRF, we used XRF‐XGT 7200 Horiba spectrometer with Rh Target X‐Ray tube, at 50 kV under vacuum. ICP‐AES measurements were performed with an Ultima‐2 spectrometer (Jobin‐Yvon Horiba). The chemical composition of the thin AZ31 Mg alloy foils after rolling was examined by Optical Emission Spectroscopy (OES) using Spectra MAXx spectrometer utilizing a Spark Analyzer MX software.

## Conflict of interest

The authors declare no conflict of interest.

## Supporting information

As a service to our authors and readers, this journal provides supporting information supplied by the authors. Such materials are peer reviewed and may be re‐organized for online delivery, but are not copy‐edited or typeset. Technical support issues arising from supporting information (other than missing files) should be addressed to the authors.

Supporting Information
