# Supplementary material for: AZ31 Magnesium Alloy Foils as Thin Anodes for Rechargeable Magnesium Batteries
Source: ChemSusChem. 2021 Aug 31;14(21):4690–6. doi: 10.1002/cssc.202101323 (PMC8596635; doi:10.1002/cssc.202101323)
Supplement: Supplementary file 1 — Supporting Information [file CSSC-14-4690-s001.pdf]

ChemSusChem

Supporting Information

AZ31 Magnesium Alloy Foils as Thin Anodes for Rechargeable Magnesium Batteries

Ananya Maddegalla, Ayan Mukherjee,* J. Alberto Blázquez, Eneko Azaceta, Olatz Leonet, Aroa R. Mainar, Aleksey Kovalevsky, Daniel Sharon, Jean-Frédéric Martin, Dane Sotta, Yair Ein-Eli, Doron Aurbach, and Malachi Noked*© 2021 The Authors. ChemSusChem published by Wiley-VCH GmbH. This is an open access article under the terms of the Creative Commons Attribution License, which permits use, distribution and reproduction in any medium, provided the original work is properly cited.

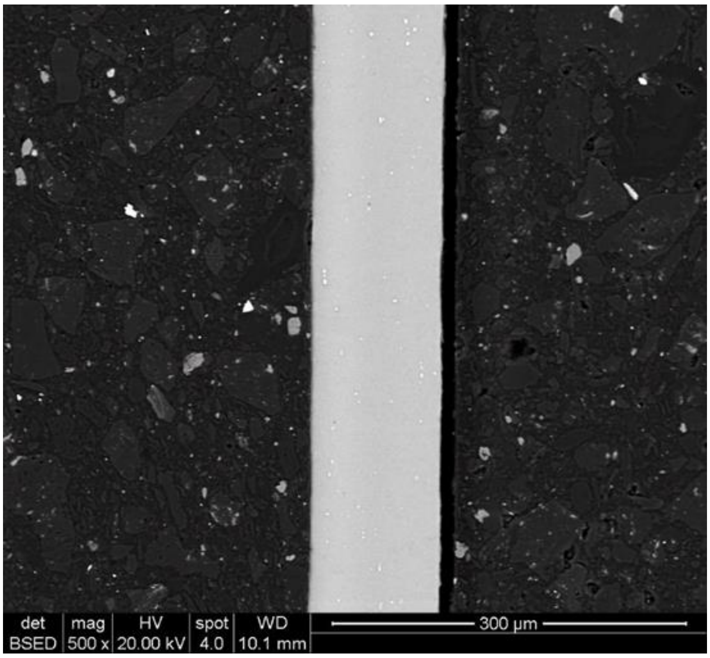

Figure S1 Cross-sectional SEM images of AZ31 100 µm

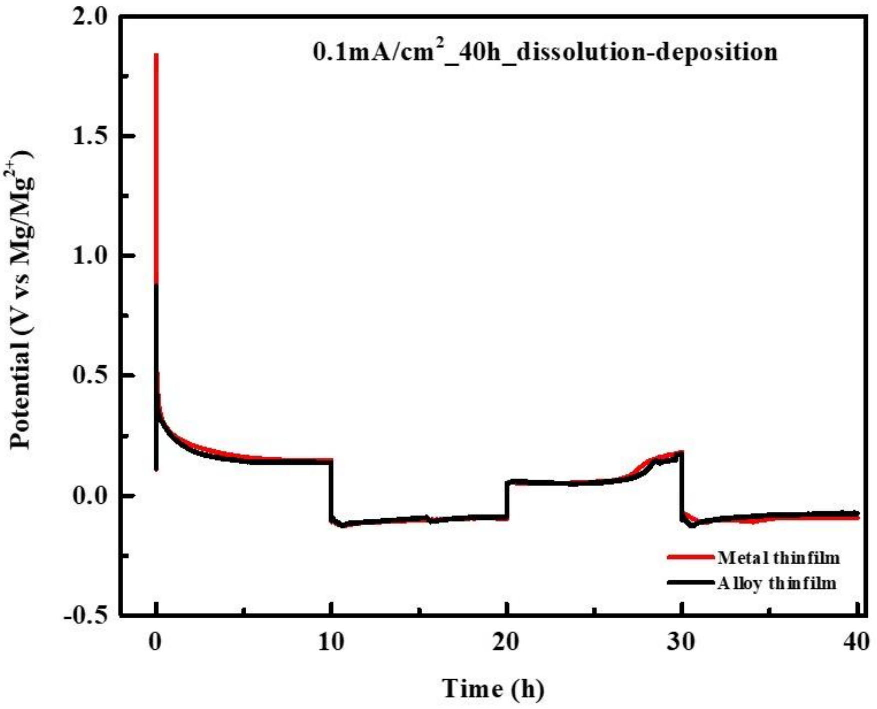

Figure S2 Voltage profile of dissolution-deposition process on AZ31 alloy thin film and Mg metal thin film as anodes at current densities of 0.1 mA/cm^2 for 40h

Rolling procedure

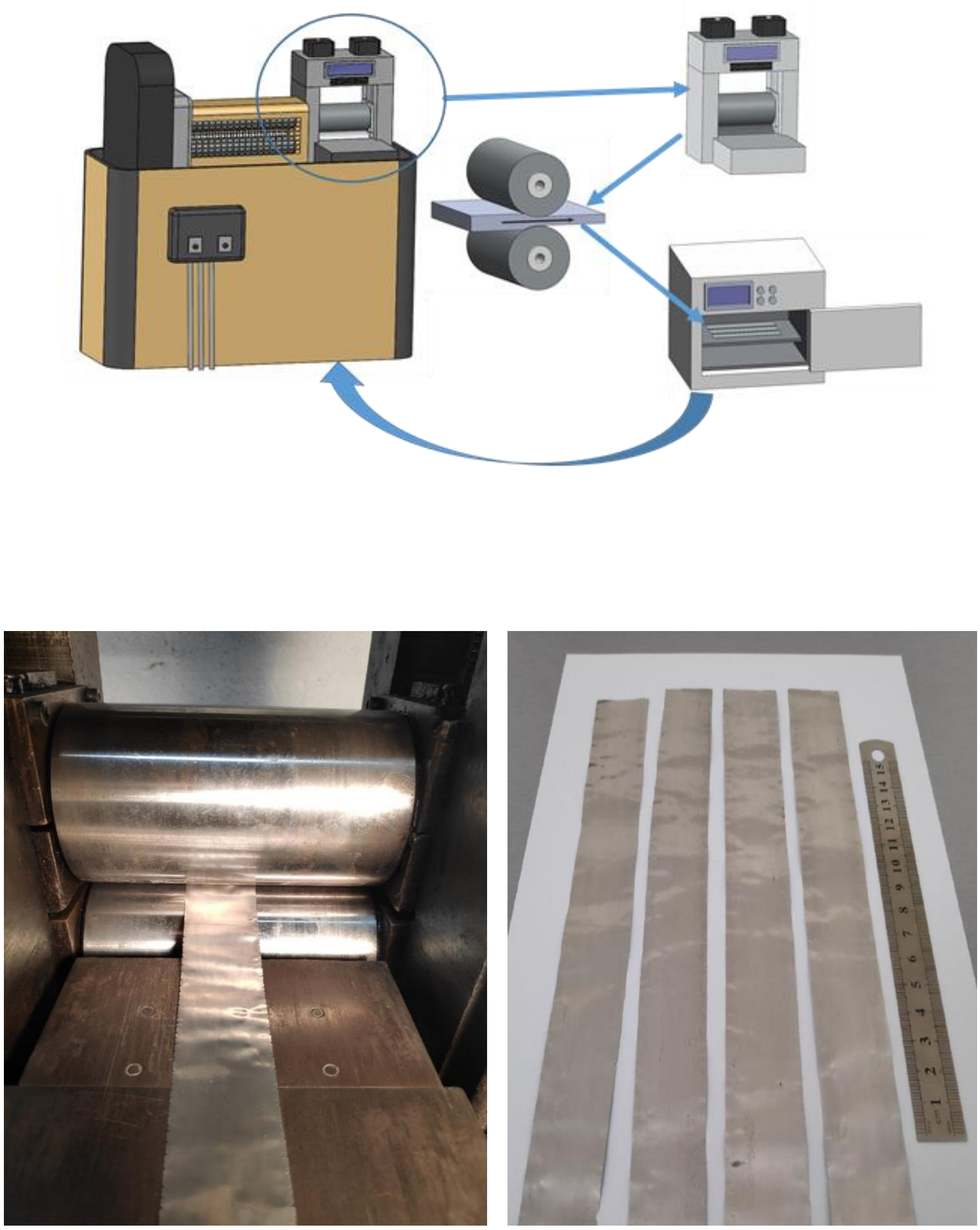

Figure S3 Scheme of the upgraded control rolling system at the Israel Metal Institute, and optical images of the AZ31 foil during the rolling process

Cross sectional SEM images of rolled AZ31 foils

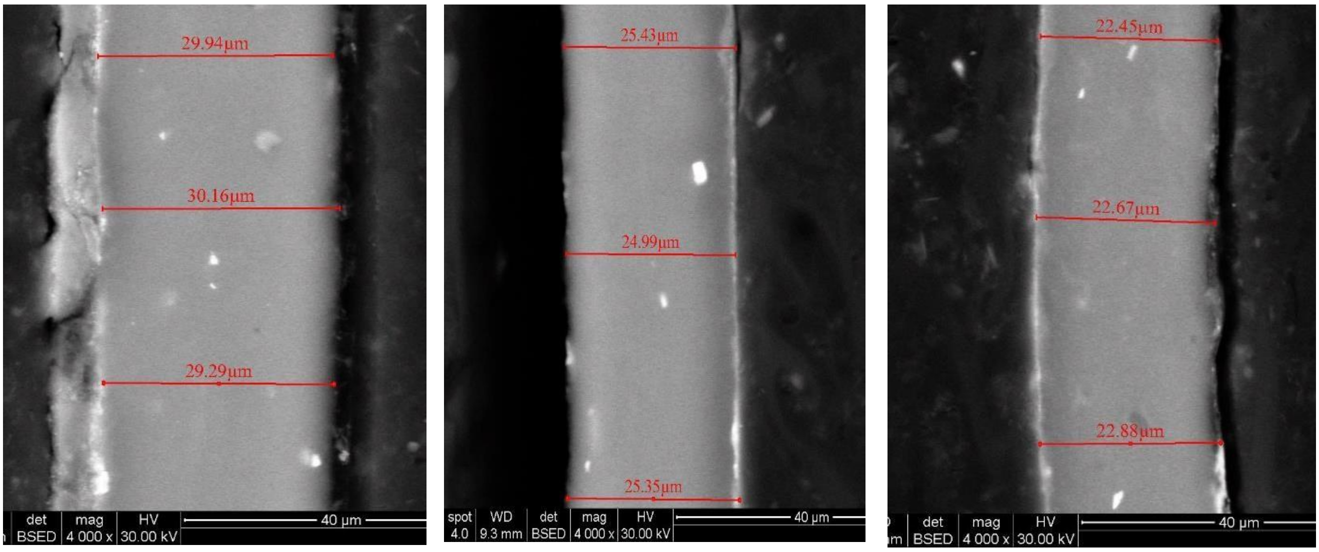

Figure S4 Cross-sectional SEM images of decreasing AZ31 foils thicknesses with increasing number of rolling processes.

Composition of the Alloy (AZ31)

Table S1. Chemical composition of AZ31 samples before rolling.

	**Al (%)**	**Zn (%)**	**Mn (%)**	**Si (%)**	**Fe (%)**	**Cu (%)**	**Ni (%)**	**Mg (%)**
↓	2.500	0.700	0.200		0.0000	0.000	0.000	
**X**	2.905	1.027	0.288	0.023	0.0047	0.001	0.002	95.7
↑	3.500	1.300	0.400		0.0050	0.100	0.030	

Table S2. Chemical composition of flat-rolled AZ31 samples

	**Al (%)**	**Zn (%)**	**Mn (%)**	**Si (%)**	**Fe (%)**	**Cu (%)**	**Ni (%)**	**Mg (%)**
↓	2.500	0.700	0.200		0.0000	0.000	0.000	
**X**	2.790	0.979	0.359	0.025	0.0044	0.001	<0.001	95.8
↑	3.500	1.300	0.400		0.0050	0.100	0.030	

Chevrel Phase (CP) cathodes preparation

CP cathodes were prepared by a standard wet coating process. A CP (Mo_6S_8 from NEI , USA) slurry was prepared using 90 wt% CP (NEI), 5 wt% carbon black (Super C45, Imerys) and 5 wt% PVDF binder (Solef® 5130, Solvay). The carbon black was dispersed into a PVDF solution (8 wt% dissolved in N-methylpyrrolidone solvent) using a high-speed disperser (Dispermat, VMA-Getzmann GmbH). Then the CP powder was added to the mixture and further dispersed until an homogeneous slurry was obtained. Finally, the slurry was refined using a three-roll mill (Exakt) to reduce the agglomerates size and avoid the presence of any defect on the final coating. The electrodes were coated on both sides of a 20 µm-thick carbon-coated nickel current collector foil (Gelon) using a comma bar roll-to-roll coating equipment in a dry room (dew point < -20 ºC). The total electrodes' loading was set at 13 mg cm^{-2}. The electrodes were dried at 50 °C for 12 hours under vacuum and then calendered to 30 % porosity.
